# Role of *yoaE* Gene Regulated by CpxR in the Survival of *Salmonella enterica* Serovar Enteritidis in Antibacterial Egg White

**DOI:** 10.1128/mSphere.00638-19

**Published:** 2020-01-08

**Authors:** Xiaozhen Huang, Mengjun Hu, Xiujuan Zhou, Yanhong Liu, Chunlei Shi, Xianming Shi

**Affiliations:** a MOST-USDA Joint Research Center for Food Safety, School of Agriculture and Biology, and State Key Lab of Microbial Metabolism, Shanghai Jiao Tong University, Shanghai, China; b Molecular Characterization of Foodborne Pathogens Research Unit, Eastern Regional Research Center, Agricultural Research Service, U.S. Department of Agriculture, Wyndmoor, Pennsylvania, USA; University of California, Davis

**Keywords:** ultrafiltration matrix, egg white, *yoaE*, *cpxR*, alkaline pH, antimicrobial peptide, *Salmonella*

## Abstract

The survival ability of Salmonella enterica serovar Enteritidis in antibacterial egg white is an important factor leading to *Salmonella* outbreaks through eggs and egg products. In this study, the role of the gene *yoaE*, encoding an inner membrane protein, in the survival of Salmonella Enteritidis in egg white, and its transcriptional regulation by CpxR were investigated. Quantitative reverse transcription-PCR (RT-qPCR) results showed that the *yoaE* gene expression was upregulated 35-fold after exposure to egg white for 4 h compared to that in M9FeS medium, and the deletion of *yoaE* (*ΔyoaE*) dramatically decreased the survival rate of bacteria in egg white to less than 1% of the wild type (WT) and the complementary strain at both 37 and 20°C, indicating that *yoaE* was essential for bacteria to survive in egg white. Furthermore, the *ΔyoaE* strain was sensitive to a 3-kDa ultrafiltration matrix of egg white because of its high pH and antimicrobial peptide components. Putative conserved binding sites for the envelope stress response regulator CpxR were found in the *yoaE* promoter region. *In vivo*, the RT-qPCR assay results showed that the upregulation of *yoaE* in a *ΔcpxR* strain in egg white was 1/5 that of the WT. *In vitro*, results from DNase I footprinting and electrophoretic mobility shift assays further demonstrated that CpxR could directly bind to the *yoaE* promoter region, and a specific CpxR binding sequence was identified. In conclusion, it was shown for the first time that CpxR positively regulated the transcription of *yoaE*, which was indispensable for survival of Salmonella Enteritidis in egg white.

**IMPORTANCE** Salmonella enterica serovar Enteritidis is the predominant *Salmonella* serotype that causes human salmonellosis mainly through contaminated chicken eggs or egg products and has been a global public health threat. The spread and frequent outbreaks of this serotype through eggs correlate significantly with its exceptional survival in eggs, despite the antibacterial properties of egg white. Research on the survival mechanisms of *S.* Enteritidis in egg white will help develop effective strategies to control the contamination of eggs by this *Salmonella* serotype and help further elucidate the complex antibacterial mechanisms of egg white. This study revealed the importance of *yoaE*, a gene with unknown function, on the survival of *S.* Enteritidis in egg white, as well as its transcriptional regulation by CpxR. Our work provides the basis to reveal the mechanisms of survival of *S.* Enteritidis in egg white and the specific function of the *yoaE* gene.

## Abstract (precis)

Salmonella enterica serovar Enteritidis is the predominant *Salmonella* serotype that causes human salmonellosis mainly through contaminated chicken eggs or egg products and has been a global public health threat. The spread and frequent outbreaks of this serotype through eggs correlate significantly with its exceptional survival in eggs, despite the antibacterial properties of egg white. Research on the survival mechanisms of *S.* Enteritidis in egg white will help develop effective strategies to control the contamination of eggs by this *Salmonella* serotype and help further elucidate the complex antibacterial mechanisms of egg white. This study revealed the importance of *yoaE*, a gene with unknown function, on the survival of *S.* Enteritidis in egg white, as well as its transcriptional regulation by CpxR. Our work provides the basis to reveal the mechanisms of survival of *S.* Enteritidis in egg white and the specific function of the *yoaE* gene.

## INTRODUCTION

Salmonella enterica serovar Enteritidis is an important foodborne pathogen that leads to human salmonellosis mainly through contaminated eggs or egg products. The main contamination site of this *Salmonella* serotype is egg white (1–3), and its survival ability in the antibacterial egg white is stronger than other *Salmonella* serotypes, as well as non-*Salmonella* bacteria such as Escherichia coli (4–6). This special ability is an important factor leading to large-scale outbreaks of salmonellosis caused by the consumption of eggs or egg products that are internally infected with *S.* Enteritidis (7–10). Thus, understanding the survival mechanisms of *S.* Enteritidis in antibacterial egg white has become an important food safety issue.

Egg white is a complex antimicrobial environment, including physical antimicrobial factors such as alkaline pH (9.3) and high viscosity, as well as biochemical antimicrobial factors such as chelators (avidin and ovotransferrin) and abundant antibacterial proteins and peptides (lysozyme, AvBD11, OvoDB1, etc.). These antimicrobial factors work together to inactivate the invading bacteria (11–13). The antibacterial properties of egg white are affected by egg storage time, temperature, and bacterial inoculum size. After fresh table eggs are stored at 37°C for 5 days or stored at 20°C for 12 days, their egg whites have comparable bacteriostatic ability and achieve maximum bactericidal capacity during storage (14). Previous studies have shown that *S.* Enteritidis mobilizes utilizes multiple mechanisms to counteract antibacterial factors in egg white. Genes or pathways involved in iron absorption (15–17), biotin synthesis (16–18), DNA replication and repair (4, 17, 19), alkaline pH adaptation (17), osmotic stress adaptation (17), envelope damage repair (including peptidoglycan biosynthesis and remodeling) (4, 17), amino acid transport and metabolism (4), lipopolysaccharide metabolism (4, 18, 20, 21), and cell motility (22) were found to be important for *S.* Enteritidis survival in egg white.

The two-component regulatory system (TCS) CpxR/A plays a central and critical role in bacterial envelop damage repair and alkaline pH adaption in egg white (16, 17). Once activated in egg white, CpxR/A upregulated genes involved in periplasmic space homeostasis, peptidoglycan cross-linking, and remodeling and envelope cross-linking to combat envelope stress caused by egg white; they also upregulated cation/antiporter *chaA* and downregulated the respiratory complexes (the *nuo* and *cyo* operons) to increase proton import and to decrease proton export, therefore maintaining bacterial pH homeostasis in the alkaline egg white (16, 17). In addition, deletion of *cpxR* gene made the survival ability of *S.* Enteritidis in egg white dramatically reduced, which further proved the importance of this TCS (17).

Through gene deletion and complementary, we demonstrated that seven differently expressed genes (including gene *yoaE*) obtained from our published transcriptome data contributed to the survivability of *S.* Enteritidis in egg white (17). Six of them (except gene *yoaE*) were included in our previous published paper (17). In this study, gene *yoaE* (*A7J12_06270*), which encodes a predicted inner membrane protein and was upregulated by 15- to 18-fold at the mRNA level in egg white (17), was further characterized. Analysis of the *yoaE* promoter region revealed conserved CpxR binding sites upstream of the *yoaE* gene, indicating that this gene was likely to be regulated by CpxR in egg white. A review of the literature indicated that only one study has been published on this gene. In *Shigella*, *yoaE* was shown to be directly upregulated by PhoP in Luria-Bertani (LB) medium, whereas no significant difference was found between wild-type and *ΔyoaE* strains in a gentamicin protection assay of HeLa cells (23). Whether the *yoaE* gene affects the survival of *S.* Enteritidis in egg white and which stress factor of egg white this gene responds to, as well as its transcriptional regulation, are all unknown.

In this study, using an egg white-resistant strain *S.* Enteritidis SJTUF 10978, we tested the importance of gene *yoaE* on the survival of *S.* Enteritidis in egg white and its ultrafiltration matrix, and we show the regulatory role of an envelope stress regulator CpxR in *yoaE* expression.

## RESULTS

### The *yoaE* gene was essential for the survival of *S.* Enteritidis in egg white.

**FIG 1:**
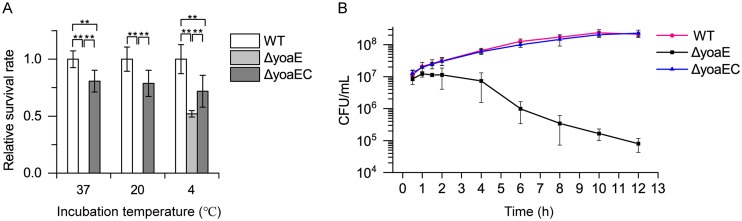
Survival of *yoaE* derivatives in egg white. (A) Survival rate of *yoaE* derivatives incubated with egg white for 24 h at 37°C and for 5 days at 20 and 4°C, respectively. (B) Growth curve of *yoaE* derivatives in egg white at 37°C for 12 h. The incubation temperatures were the same as the egg storage temperature. Mean values were taken from three independent experiments, and the standard errors of the means (error bars) are shown. **, *P* < 0.01.

In order to determine the importance of *yoaE* gene on the survival ability of *S.* Enteritidis in egg white, the relative survival rates of *yoaE* derivatives (*ΔyoaE* and *ΔyoaEC*) at 37°C (lab cultivation temperature), 20°C (the egg storage temperature in some food enterprises in China), and 4°C (refrigerator temperature) were tested. As shown in Fig. 1A, compared to the WT and the complementary strain (*ΔyoaEC*), the survival ability of the *ΔyoaE* strain significantly decreased under all three incubation conditions. Moreover, upon incubated with egg white at 37°C for 24 h and at 20°C for 5 days, the survival rates of *ΔyoaE* strain were less than 1% of the WT and *ΔyoaEC* strains, whereas the *ΔyoaE* strain showed only a 50% reduction in bacterial concentration compared to the WT when incubated with egg white at 4°C. In addition, growth curve studies on *yoaE* derivatives in egg white stored at 37°C showed that the *ΔyoaE* strain grew at the beginning 2 h and then was gradually inactivated by egg white, whereas the WT and *ΔyoaEC* strains presented growth since inoculation (Fig. 1B). Further, expression of *yoaE* gene was also proven to be upregulated 35-fold in the WT strain after incubation with egg white for 4 h compared to incubation in M9FeS medium (see Fig. 3). These results indicated that *yoaE* played an important role in the survival of *S.* Enteritidis in egg white under industrial storage conditions and higher temperatures.

### Alkaline pH and antimicrobial peptides of 3-kDa ultrafiltration matrix from egg white rendered the *ΔyoaE* strain sensitive.

**FIG 2:**
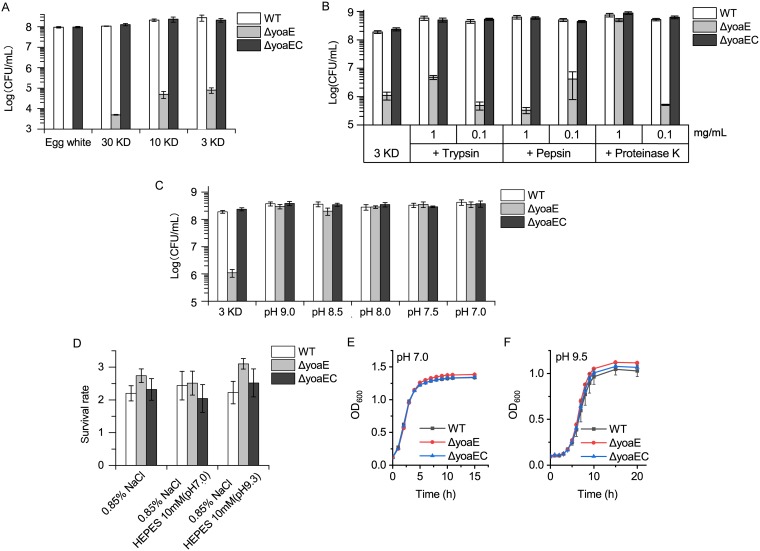
Survival of *yoaE* derivatives in egg white and its ultrafiltration matrix. (A) Survival of the *ΔyoaE* strain in egg white and its ultrafiltration matrix with different molecular weight cutoffs. (B) Survival of *ΔyoaE* strain in a 3-kDa ultrafiltration matrix digested by different proteases. (C) Survival of a *ΔyoaE* strain in 3-kDa ultrafiltration matrix of different pHs. (D) Viability of *yoaE* derivatives in physiological saline of different pHs. (E) Growth curve of *yoaE* derivatives in neutral-pH LB medium. (F) Growth curve of *yoaE* derivatives in alkaline-pH LB medium (9.5).

In order to identify the egg white components that made *ΔyoaE* strain sensitive, the survival ability of *yoaE* derivatives in egg white ultrafiltration matrices of 30, 10, and 3 kDa were tested. As shown in Fig. 2A, compared to the WT and complementary strains, the *ΔyoaE* strain was sensitive to all the three ultrafiltration matrixes, and the survival ability of the *ΔyoaE* strain increased as the matrix components became simpler. This result indicated that the 3-kDa ultrafiltration matrix itself played an antibacterial effect against the *ΔyoaE* strain, and the other antibacterial proteins in egg white enhanced the antibacterial effect. Here, we focused on the antibacterial mechanism of 3-kDa ultrafiltration matrix against the *ΔyoaE* strain.

The 3-kDa ultrafiltration matrix presents an alkaline pH (9.5) and may contain antibacterial peptides of <27 amino acids (aa). To determine whether the alkaline pH and peptides participate in the antibacterial ability of the 3-kDa ultrafiltration matrix, we tested the survival ability of *yoaE* derivatives in 3-kDa ultrafiltration matrix digested by different proteases (Fig. 2B) and with different pHs (Fig. 2C). The results showed that 1 mg/ml trypsin and pepsin did not abolish the antibacterial activity of the 3-kDa ultrafiltration matrix against *ΔyoaE* strain, whereas 1 mg/ml proteinase K eradicated the antibacterial activity. This result suggested that peptides participated in the antibacterial ability of 3- kDa ultrafiltration matrix, and they were sensitive to proteinase K.

Decreasing the pH of the 3-kDa ultrafiltration matrix, even if only by 0.5, would cause the 3-kDa ultrafiltration matrix to lose its antibacterial ability against the *ΔyoaE* strain (Fig. 2C). However, the *ΔyoaE* strain was not sensitive to alkaline pH (9.5) itself, regardless of whether it was cultured in oligotrophic saline (Fig. 2D) or in eutrophic LB medium (Fig. 2E and F), since under these growth conditions no difference was found between the WT and *ΔyoaE* strains. This showed that the *yoaE* deletion strain was sensitive to 3-kDa ultrafiltration matrix of egg white because of both its alkaline pH and antibacterial peptides.

### CpxR positively regulated the expression of gene *yoaE*.

**FIG 3:**
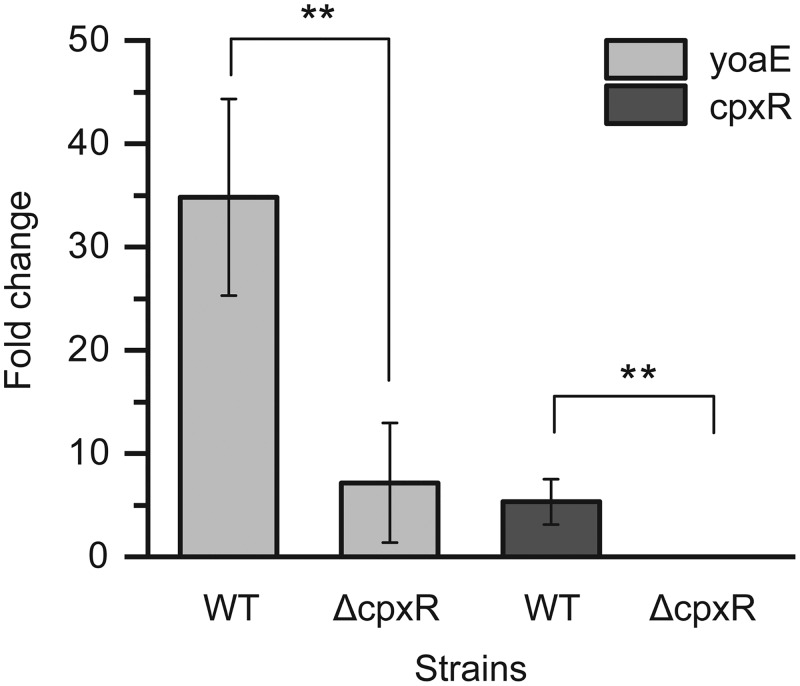
Relative expression changes of genes *yoaE* and *cpxR* at mRNA level in egg white compared to that of M9FeS medium in both wild-type and *ΔcpxR* strains. The expression of *yoaE* and *cpxR* gene in the single strain in M9FeS was set to 1. Mean values are taken from three independent experiments, and the standard errors of the means (error bars) are shown. **, *P* < 0.01.

Analysis of the *yoaE* promoter region with Virtual Footprint (http://www.prodoric.de/vfp/vfp_promoter.php) revealed the presence of two probable partially overlapping CpxR binding sites located at approximately bp −235 to −262 (The translation start site is defined as +1) of its promoter region (see Fig. S1 in the supplemental material). In order to determine whether CpxR regulates *yoaE* in egg white *in vivo*, we compared the *yoaE* gene expression level between the WT and the *cpxR* deletion mutant (*ΔcpxR*) using RT-qPCR assays (Fig. 3). The results showed that the *yoaE* gene was induced in both *ΔcpxR* and WT strains in egg white after incubation with egg white for 4 h. However, the increase in the *ΔcpxR* strain (7-fold) was just one-fifth that in the WT strain (35-fold). Meanwhile, we found that *cpxR* was also induced 5-fold in egg white at 4 h. This result indicates that CpxR positively regulated the expression of *yoaE* gene in egg white.

10.1128/mSphere.00638-19.1 FIG S1 Analysis of *yoaE* promoter region. CpxR binding sites was predicted by Virtual Footprint (http://www.prodoric.de/vfp/vfp_promoter.php). TSS, transcriptional starting site of *yoaE* gene according to the strand-specific RNA sequencing result of SJTUF 10978 in egg white (GEO accession no. GSE113880). yoaEPF and yoaEPR are primers used for amplification of *yoaE* promoter sequence that used in DNase I footprinting. Download FIG S1, TIF file, 0.9 MB. Copyright © 2020 Huang et al. 2020 Huang et al. This content is distributed under the terms of the Creative Commons Attribution 4.0 International license.

**FIG 4:**
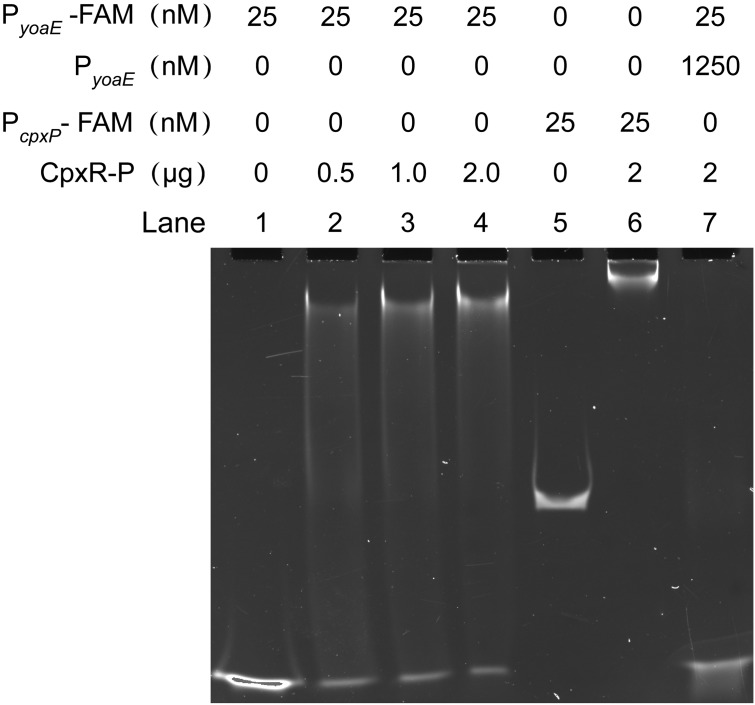
Verification of the directly binding of CpxR-P to the *yoaE* promoter regions by EMSA. Lanes 1 to 4, FAM-labeled *yoaE* Promoter (P*_yoaE_*-FAM) incubated with different amounts of phosphorylated CpxR (CpxR-P, 0, 0.5, 1.0, and 2 μg); lanes 5 and 6, FAM-labeled *cpxP* promoter (P*_cpxP_*-FAM) incubated without (lane 5) or with (lane 6) CpxR-P, with P*_cpxP_*-FAM used as a positive control; lane 7, 50× unlabeled *yoaE* promoter probe (P*_yoaE_*) competed with P*_yoaE_*-FAM. For every lane, 0.5 μg of poly(dI-dC) was added to decrease nonspecific binding.

**FIG 5:**
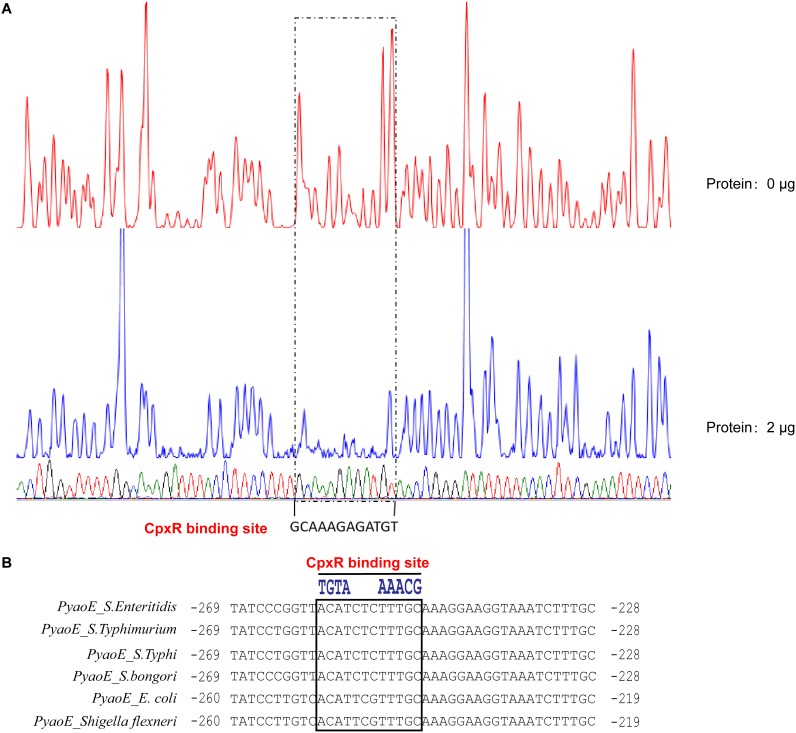
Identification of phosphorylated CpxR binding sequence in *yoaE* promoter and alignment analysis in different species. (A) DNase I footprinting assays of phosphorylated CpxR binding sequence on *yoaE* promoter region. A 250-bp FAM-labeled *yoaE* promoter DNA fragment was incubated without or with 2 μg pf phosphorylated CpxR (CpxR-P) and then subjected to DNase I digestion and fragment length analysis. The fluorescence signal of the labeled DNA fragments is plotted against the sequence of the fragment. The sequence of protected region bound by CpxR-P is shown. (B) Alignment of CpxR binding site on *yoaE* promoter region in the investigated species.

In order to determine whether this regulatory relationship is direct or indirect, as well as the specific binding sites, electrophoretic mobility shift assays (EMSAs) and DNase I footprinting assays were performed *in vitro*. The EMSA results showed that phosphorylated CpxR protein (CpxR-P) could bind to the promoter of its known regulatory target—the FAM-labeled *cpxP* promoter probe (P*_cpxP_*-FAM)—and produce a migration band, indicating that the experimental system worked well. Further, CpxR-P could bind to the 50-bp FAM-labeled *yoaE* promoter probe (P*_yoaE_*-FAM) and produced a migration band (Fig. 4). The signal intensity of the migration band increased as the CpxR protein concentration increased, and the amount of free probe decreased accordingly. The binding of P*_yoaE_*-FAM by CpxR-P was completely inhibited by 50× unlabeled *yoaE* promoter probe (P*_yoaE_*), indicating that the binding of CpxR-P to *yoaE* promoter was specific (Fig. 4). The specific CpxR-P binding site on *yoaE* promoter was also identified by DNase I footprinting analysis with 153-bp *yoaE* promoter sequence (Fig. 5A). Comparison of sequencing peak maps of samples without or with 2 μg of CpxR-P protein revealed a conserved 12-nucleotide protected region (GCAAAGAGATGT) which was part of the predicted CpxR binding site 1 (see Fig. S1 in the supplemental material; Fig. 5A).

Analysis of the *yoaE* promoter region in several investigated species revealed that this identified CpxR binding site was conserved in S. bongori and S. enterica, including *S.* Enteritidis, *S.* Typhimurium, and *S*. Typhi, as well as E. coli and Shigella flexneri, indicating that this regulatory mechanism is conserved in these species (Fig. 5B). Surprisingly, although the predicted CpxR binding site 2 (see Fig. S1 in the supplemental material) was also conserved in these species, it was not identified by DNase I footprinting analysis.

## DISCUSSION

In this study, we found that the expression of inner membrane protein gene *yoaE* was induced in egg white and was essential for *S.* Enteritidis survival in egg white and its 3-kDa ultrafiltration matrix. In addition, we also demonstrated the directly positive regulation role of the envelope stress regulator CpxR on *yoaE* gene expression both *in vivo* and *vitro* and identified the precise CpxR binding site on the *yoaE* promoter.

Our results showed that, although egg white 3-kDa ultrafiltration matrix did not contain known egg white bacteriostatic proteins, such as lysozyme and ovotransferrin, it did inhibit the growth of the *yoaE* gene deletion mutant. There are two possible mechanisms for this inhibition. The first is that the inhibition is caused by small antimicrobial peptides which require alkaline pH condition to function. The second possible mechanism is that the inhibition effect might be produced by the synergistic action of the alkaline pH and the antimicrobial peptide. Further confirmation of the inhibition mechanism requires qualitative and quantitative determinations of the types and amounts of antimicrobial peptides in the 3-kDa matrix. The antimicrobial peptides may be low molecular weight and active degradation products of known antibacterial peptides or proteins, such as β-defensins AvBD11, gallin (OvoDA1 and OvoDB1), lysozyme, and ovotransferrin, during egg storage. The short peptide degradation products of lysozyme have been shown to have antibacterial activity (24–26).

The second possible inhibition mechanisms of the 3-kDa matrix toward *yoaE* deletion strain can be explained by our published transcriptome data of *S.* Enteritidis survival in egg white (17). The expression of *phoP*-*phoQ* and *pmrA*-*pmrB*, two critical two-component regulatory systems (TCSs) that regulate bacterial resistance to antimicrobial peptides, was dramatically decreased in egg white (17). These two TCSs have been shown to induce and function under acidic conditions, and their expression and function are inhibited in an alkaline pH environment (27–31). Therefore, the alkaline pH of egg white can inhibit the expression and function of these two TCSs, thereby inhibiting the resistance of bacteria to antimicrobial peptides. Thus, *yoaE* deletion strain may be sensitive to the synergistic bacteriostatic action of alkaline pH and antimicrobial peptides.

**FIG 6:**
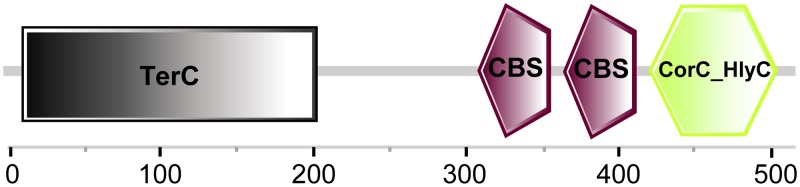
YoaE protein structure predicted by SMART (http://smart.embl-heidelberg.de/).

The presence of the *yoaE* gene causes bacteria to develop inherent resistance to the 3-kDa matrix and the whole egg white. However, revelation of this resistance mechanism requires further determination of the *yoaE* function, which is as yet still unclear. CpxR/A is a critical TCS contributing to the survival of *S.* Enteritidis in egg white and functions by extensively regulating genes that help bacteria resist envelope stress and alkaline pH (16, 17). In the present study, we proved that *yoaE* gene is a CpxR/A regulon and that this regulatory relationship should be conserved in neighboring species, indicating that *yoaE* should be envelope stress-related gene, which provides clues to the *yoaE* gene function. On the other hand, prediction of the YoaE protein structure by SMART (Fig. 6) revealed that *yoaE* is an inner membrane protein with a TerC domain (14 to 204 aa) at the N terminus and two CBS domains (309 to 419 aa) and a CorC_HlyC domain (431 to 512 aa) at the C terminus, indicating that this gene is probably involved in ion transport (32, 33). However, the kinds of ions transported by the YoaE protein and how this predictive ion transporter is involved in egg white resistance require further study.

Our results add to our understanding of the survival mechanism of *S.* Enteritidis in egg white and provide further information on the function of the *yoaE* gene and the CpxR regulon.

## MATERIALS AND METHODS

### Bacterial strains and growth media.

The complete list of bacterial strains used in this study is in Table S1. An egg white-resistant strain *S.* Enteritidis, SJTUF10978, and its gene derivatives were used in this study (17). M9FeS (M9 minimal medium supplemented with 2 mg/liter FeCl_3_⋅6H_2_O and microelements) (17) or Luria-Bertani (LB) broth was routinely used in this study, and 37°C was the routine culture temperature without special instructions. Chloramphenicol (25 μg/ml) or ampicillin (100 μg/ml) was added when needed.

10.1128/mSphere.00638-19.2 TABLE S1 Strains and plasmids used in this study. Download Table S1, DOCX file, 0.02 MB. Copyright © 2020 Huang et al. 2020 Huang et al. This content is distributed under the terms of the Creative Commons Attribution 4.0 International license.

### Preparation of egg white and its ultrafiltration matrix.

In this study, fresh unfertilized specific-pathogen-free (SPF) eggs that laid by 20- to 22-week-old chickens were purchased from Beijing Merial Vital Laboratory Animal Technology (Beijing, China) and used within 2 days after laying. Preparation of egg white stored at 37, 20, or 4°C was done as described previously (17). The final prepared egg white was stored at 4°C for up to 5 days. The egg white ultrafiltration matrixes were prepared with egg white stored at 37°C for 5 days. The egg white was centrifuged with a 30-kDa ultrafiltration tube (Millipore), and the filtrate was called 30-kDa ultrafiltration matrix. The 10-kDa ultrafiltration matrix was obtained by centrifuging the 30-kDa ultrafiltration matrix with 10-kDa ultrafiltration tube (Millipore), and the 3-kDa ultrafiltration matrix was obtained by centrifuging the 10-kDa ultrafiltration matrix with 3-kDa ultrafiltration tube (Millipore). This centrifugation was performed at 4°C. Ultrafiltration matrices with different molecular weight cutoffs were stored at –80°C before use.

### Antibacterial experiments with egg white and its ultrafiltration matrix.

Egg white antibacterial experiments were performed as described previously (17). Briefly, fresh bacteria (1 ml) of logarithmic-phase culture were harvested, washed twice, and resuspended with phosphate-buffered saline (10 mM Na_2_HPO_4_, 1.8 mM KH_2_PO_4_, 137 mM NaCl, 2.7 mM KCl [pH 7.4]). The bacterial suspensions were diluted and then inoculated into egg white to achieve a final inoculum concentration of 5 × 10^6^ CFU/ml and a final egg white concentration of 80% (vol/vol) in a Falcon 96-well microplate. Routinely, the mixtures were then incubated at 37°C (RH 65%) for 24 h. For Fig. 1A, the mixtures were also incubated with egg white at 20 or 4°C, respectively, for 5 days. The inoculation concentration and the final bacterial concentration after incubation were determined by serial dilution plate counts. The relative survival rate of each strain was calculated by dividing the final concentration of the bacteria by the initial inoculum concentration.

The 3-kDa ultrafiltration matrix digestion experiments with different proteases were performed by adding proteases to different final concentrations, followed by incubation at 37°C for 2 h. The pH of the 3-kDa ultrafiltration matrix was adjusted by using a pH meter with HCl. The pH of the saline was buffered using 10 mM HEPES. The survival ability of bacteria in a 3-kDa ultrafiltration matrix either digested by proteases or with different pHs or in saline with different pHs was assessed using egg white antibacterial experiments. The experiments were performed as three independent biological replicates, using three technical repeats for each biological repeat. Significance analysis was calculated using one-way analysis of variance, and a *P* value of 0.05 was considered significant.

### Growth study of bacteria.

Growth curves of bacteria in egg white were measured as described previously (17). Briefly, fresh mid-log-phase bacterial cultures in M9FeS medium were washed and inoculated into 50 ml of egg white with a final inoculum concentration of 5 × 10^6^ CFU/ml and a final egg white concentration of 80% (vol/vol). The culture was mixed and incubated at 37°C under a 65% humidified atmosphere. The inoculation concentration and bacterial concentrations at 0.5, 1, 2, 4, 6, 8, 10, and 12 h after inoculation were determined by serial dilution plate counting. Data from three independent biological replicates were used (means ± the standard deviations). Growth curve measurement of WT and *yoaE* gene derivatives in LB medium at pH 7.0 and 9.5 were performed at 37°C using Bioscreen C (OY Growth Curves, Finland). Three independent experiments were carried out for each strain.

### RNA isolation.

To prepare RNA samples of WT and *ΔcpxR* strains, bacterial culture and inoculation of the egg white were conducted as described above for the growth study of bacteria in egg white. Mid-log-phase bacterial cultures in M9FeS medium (0 h sample) were inoculated into egg white for 4 h (4-h sample) and taken for RNA extraction. Ice-cold RNA-stabilizing solution was used to stabilize mRNA in bacterial samples, and TRIzol reagent (Invitrogen) was used for RNA extraction. The detailed method for bacterial culture, sampling, and RNA extraction is described elsewhere (17). Three independent biologically repeated experiments were performed at different times with different batches of SPF eggs.

### Quantitative RT-PCR assays.

The specific experimental method is presented elsewhere (17). The expression of 16S rRNA was used as an internal reference. Primers for the *cpxR*, *yoaE*, and 16S rRNA genes used in qPCR are listed in Table S2 in the supplemental material. Both removal of the residual genomic DNA and cDNA synthesis were performed using a PrimeScript RT reagent kit with a gDNA eraser (DRR047A; TaKaRa, Dalian, China) according to the manufacturer’s instructions. TB Green Premix Ex Taq II reagent (TaKaRa) was used for quantitative PCR amplification. The amplification efficiencies of the primers were between approximately 0.94 and 0.95, and the relative transcriptional level was determined using the Pfaffl method (34).

10.1128/mSphere.00638-19.3 TABLE S2 Primers used in this study. Download Table S2, DOCX file, 0.02 MB. Copyright © 2020 Huang et al. 2020 Huang et al. This content is distributed under the terms of the Creative Commons Attribution 4.0 International license.

### Bacterial genetic manipulations.

The strains, plasmids, and oligonucleotide primers used are listed in Tables S1 and S2 in the supplemental material. A bacteriophage λ Red recombination system (35) was used to construct gene deletion mutants of *S.* Enteritidis SJTUF10978, and the suicide plasmid pRE112 (36) was used to construct complementary strains. The specific experimental details were described by Huang et al. (17). The accuracy of deletion mutants and complementary strains was verified by PCR and sequencing.

### Construction of CpxR expression plasmid and purification of His-tagged CpxR protein.

The *cpxR* gene CDS was amplified with the primers cpxRF1 and cpxRR699 (Table S2) and then cloned into pET28a with NdeI and XhoI sites to generate the expression plasmid pET28a-HisCpxR. The final plasmid was confirmed by sequencing. The N-terminal His-tagged CpxR protein was purified from E. coli BL21(DE3)pLysS with His-Select nickel affinity gel (Ni-NTA beads 6FF; Smart-Lifesciences) according to the manufacturer’s instructions. The purified protein presented a single band on SDS-PAGE. The CpxR protein was then desalted and concentrated using a 10-kDa Milipore ultracentrifugal filter at 4°C. The concentrated protein was divided into small portions and stored at –80°C before use. The CpxR protein concentration was measured with a BCA protein assay kit (Tiangen, China).

### Electrophoretic mobility shift assay.

Purified N-terminal His-tagged CpxR proteins were phosphorylated using acetyl phosphate lithium potassium salt (sigma) similar as previously described (37). Briefly, purified CpxR was incubated with 50 mM acetyl phosphate lithium potassium salt in a reaction buffer containing 50 mM Tris-HCl (pH 8.0), 2 mM MgCl_2_, 125 mM KCl, and 10% glycerin at 30°C for 2 h. A 50-bp FAM-labeled *yoaE* promoter probe (P*_yoaE_*-FAM) or unlabeled *yoaE* promoter probe (P*_yoaE_*) was generated by annealing two complementary primer sequences in 1× TEN buffer (10 mM Tris-Cl [pH 8.0], 1 mM EDTA [pH 8.0], 100 mM NaCl) containing 5 mM MgCl_2_ with a PCR instrument. A 164-bp FAM-labeled *cpxP* promoter probe (P*_cpxP_*-FAM) was generated by PCR amplification using the primers cpxPF1 and cpxPR1-FAM, and then the probe was purified by using a AxyPrep PCR cleanup kit. The primer sequences are listed in Table S2 in the supplemental material. The binding reactions were performed in a 20-μl system containing 50 mM Tris (pH 8.0), 125 mM KCl, 8% glycerin, 2 mM MgCl_2_, 0.01 mg/ml bovine serum albumin, 1 mM dithiothreitol, 0.5 to 2 μg of phosphorylated CpxR (CpxR-P), 25 nM FAM-labeled probes, and 0.5 μg of poly(dI-dC), with or without 1,250 nM (50×) competitor probe. The reaction systems were incubated at 30°C for 30 min. Native polyacrylamide gels (5.5%) were preelectrophoresed for 1 h, and then the samples were loaded and separated by electrophoresis using an ice bath and scanned by using a ChemiDoc touch imaging system (Bio-Rad, Hercules, CA).

### DNase I footprinting assay.

The 153-bp *yoaE* promoter sequence was amplificated by using the primers yoaEPF and yoaEPR (Table S2 and Fig. S1 in the supplemental material) from the *S.* Enteritidis SJTUF10978 genome DNA and then cloned into the pMD19T vector to generate plasmid p19T-PyoaE. To prepare fluorescent FAM-labeled probes, the promoter region was PCR amplified from plasmid p19T-PyoaE using the primers of M13F (FAM) and M13R. The FAM-labeled probes were purified by using the Wizard SV gel and PCR Clean-Up system (Promega) and quantified with a NanoDrop 2000C (Thermo Fisher, Inc., Wilmington, DE).

DNase I footprinting assays were performed essentially as described by Wang et al. (38). For each assay, 250 ng of *yoaE* promoter probes was incubated with 0 or 2 μg of CpxR-P and 4 μg of salmon sperm DNA in a total volume of 40 μl (the same buffer used for the EMSA). After incubation for 30 min at 30°C, 10 μl of solution containing about 0.015 U of DNase I (Promega) and 100 nmol of freshly prepared CaCl_2_ was added, and further incubation was performed at 37°C for 1 min. The reaction was stopped by adding 140 μl of DNase I stop solution (200 mM unbuffered sodium acetate, 30 mM EDTA, and 0.15% SDS). The samples were first extracted with phenol-chloroform and then precipitated with ethanol. The pellets were dissolved in 30 μl of H_2_O. Preparation of the DNA ladder, electrophoresis, and data analysis were performed as described previously (38), except that the samples were analyzed with an ABI 3500 DNA sequencer (Applied Biosystems).

### Analysis of *yoaE* promoter in different species.

The promoter region of *yoaE* (*A7J12_06270*) in SJTUF 10978 and its homologous genes from different species, including STM 1828 from Salmonella enterica serovar Typhimurium strain LT2 (GenBank accession number AE006468.2), STY1958 from Salmonella enterica serovar Typhi strain CT18 (GenBank accession number AL513382.1), b1816 from Escherichia coli strain K-12 substrain MG1655 (GenBank accession number CP032667.1), SBG_1685 from Salmonella bongori NCTC 12419, and SF1412 from Shigella flexneri 2a strain 301 (GenBank accession number AE005674.2), were obtained from the National Center for Biotechnology Information and aligned using the software Molecular Evolutionary Genetics Analysis (MEGA), and the region with the identified CpxR binding site was illustrated.
