# Supplementary figures and images for: Role of yoaE Gene Regulated by CpxR in the Survival of Salmonella enterica Serovar Enteritidis in Antibacterial Egg White
Source: mSphere. 2020 Jan 8;5(1):e00638-19. doi: 10.1128/mSphere.00638-19 (PMC6952189; doi:10.1128/mSphere.00638-19)

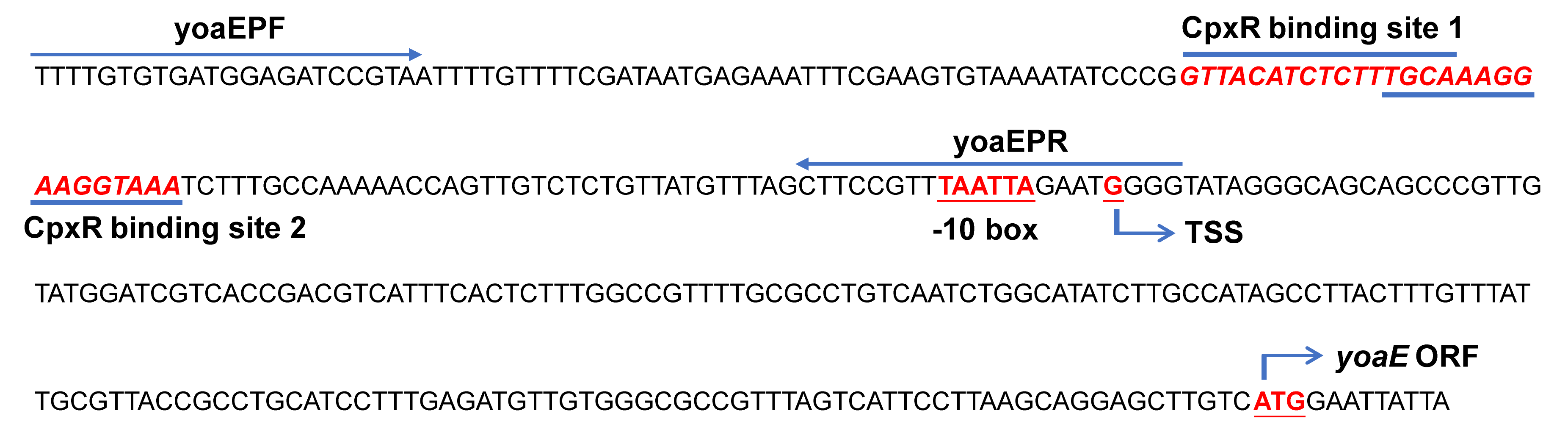

Supplement: FIG S1 [file mSphere.00638-19-sf001.tif]
