# Supplementary material for: Role of yoaE Gene Regulated by CpxR in the Survival of Salmonella enterica Serovar Enteritidis in Antibacterial Egg White
Source: mSphere. 2020 Jan 8;5(1):e00638-19. doi: 10.1128/mSphere.00638-19 (PMC6952189; doi:10.1128/mSphere.00638-19)
Supplement: TABLE S1 [file mSphere.00638-19-st001.docx]

**Table S1 Strains and plasmids used in this study**

| **Strain and plasmid** | **Description or relevant genotype** | **Reference** |
| --- | --- | --- |
| SJTUF 10978 | *Salmonella* enterica Serovar Enteritidis strain, high survival ability in albumen | (1) |
| SJTUF 10978 *ΔyoaE* | Deletion mutant of *yoaE* (*A7J12_06270*), encoding a function unknown inner membrane protein | This work |
| SJTUF 10978 *ΔyoaEC* | Complementary strain of *ΔyoaE* | This work |
| SJTUF 10978 *ΔcpxR* | Deletion mutant of *cpxR* (*A7J12_20900*), response regulator of the two-component regulatory system CpxA/CpxR | This work |
| *E. coli* SM10 λ *pir* | *thi, thr leu tonA lacY supE recA::RP4-2-Tc::Mu* Km^r^ λ *pir*, amplify plasmid pKD3 and pRE112 | Laboratory stock |
| DH5α | *F-*, *endA1*, *thi-1*, *recA1*, *Φ80lacZ*, *Δ M15* | Laboratory stock |
| BL21（DE3）pLysS | *F^-^*, *ompT*, *hsdS*(*r_B_^-^m_B_^-^*), *gal*, *dcm*(*DE3*) pLysS, Cm^r^ | Laboratory stock |
| plasmid pKD46 | Expresses bacteriophage λ Red recombinase, Amp^r^ | Gift from Professor Yao |
| plasmid pKD3 | Source for chloramphenicol acetyltransferase cassette, *pir* dependent, Amp^r^ | Gift from Professor Yao |
| plasmid pCP20 | Expresses FLP recombinase, Amp^r^ | Gift from Professor Yao |
| plasmid pRE112 | pGP704 suicide plasmid, *pir* dependent, *oriT*, *oriV*, *sacB*, Cm^r^ | (2) |
| Plasmid pET28a | Expression vector containing His tag, Km^r^ | Laboratory stock |
| Plasmid pET28a-HisCpxR | pET28a vector ligated with *cpxR* gene with NdeⅠ and XhoⅠ site, Km^r^ | This work |
| Plasmid pRE112*yoaEC* | pRE112 derivative containing gene *yoaE* and its homologous arm fragment, cm^r^;constructed with SmaⅠ and XbaⅠ | This work |
| pMD19T | TA cloning vector | Purchased from Takara |
| Plasmid p19T-PyoaE | pMD-19T ligated with 153 bp *yoaE* promoter sequence | This work |

**Km, kanamycin; Cm, chloromycetin; Amp, ampicillin.**

**Reference**

1. Huang X, Zhou X, Jia B, Li N, Jia J, He M, He Y, Qin X, Cui Y, Shi C, Liu Y, Shi X. 2019. Transcriptional Sequencing Uncovers Survival Mechanisms of *Salmonella* *enterica* Serovar Enteritidis in Antibacterial Egg White. mSphere 4:1–19.

2. Edwards RA, Keller LH, Schifferli DM. 1998. Improved allelic exchange vectors and their use to analyze 987P fimbria gene expression. Gene 207:149–157.
