# Supplementary material for: Role of yoaE Gene Regulated by CpxR in the Survival of Salmonella enterica Serovar Enteritidis in Antibacterial Egg White
Source: mSphere. 2020 Jan 8;5(1):e00638-19. doi: 10.1128/mSphere.00638-19 (PMC6952189; doi:10.1128/mSphere.00638-19)
Supplement: TABLE S2 [file mSphere.00638-19-st002.docx]

| **Table S2 Primers used in this study** | | | | |
| --- | --- | --- | --- | --- |
| **Primer name** | **Sequence （5'-3'）*^a^*** | **Target gene** | **Application** |  |
| 16SrDNAF580 | CGAATTAAACCACATGCTCCAC | *16S rDNA* | qPCR |  |
| 16SrDNAR746 | CTGGTAGTCCACGCCGTAAAC |  |  |  |
| cpxRF497 | ATTTGCTGGCGCAACACC | *cpxR* | qPCR |  |
| cpxRR683 | ATCAGATAGCCGCGACCAC |  |  |  |
| yaoEF644 | CGCGGCAATTGGTTTCTC | *yaoE* | qPCR |  |
| yoaER873 | GCCGTTAATCATGTAGCGTTCT |  |  |  |
| yoaEF46m | GCCGTTTAGTCATTCCTTAAGCAGGAGCTTGTCATGGAATTATTAAGTGTAGGCTGGAGCTGCTTCG | *yoaE* | Construct *yoaE* mutant via λ Red recombination |  |
| yoaER1603 | GTCCGCGATGCCGCATCTATAGACTTGATTCCTGCGTGTGACGTCACATATGAATATCCTCCTTAG |  |  |  |
| yoaEF147m | TCACCGACGTCATTTCACTCTT | *yoaE* | Verification of the target gene mutation |  |
| yoaER1674 | CCCATTAGCGAATTTCCACAG |  |  |  |
| PpKD3F951 | AAGTGATCTTCCGTCACAGGTAG | localized in the Cm^r^ fregment of pKD3 | Verification of the target gene mutation |  |
| PpKD3R258 | CCATGGGCAAATATTATACGCA |  |  |  |
| yoaEF822m1 | AGTGCGACCAGCTCGTCA | *yoaE* | Amplification of the *yoaE* gene and its upstream and Downstream homology arms |  |
| yoaER836 | CTCTAGCTTCTAGACAACGGCCAGCAGAATGA (XbaⅠ) |  |  |  |
| PpRE112F3047 | TTTTCTGGTGCGTACCGGGT | localized at upstream of the multiple cloning sites of plasmid pRE112 | Verification of the recombinant plasmid pRE112yoaEC |  |
| cpxRF1 | CTCTAGCTCATATGAATAAAATCCTGTTAGTTGATGA (NdeⅠ) | *cpxR* | Amplification of *cpxR* CDS and construction of its expression vector |  |
| cpxRR699 | CTCTAGCTCTCGAGCTATCATGAAGCGGAAACCATC (XhoⅠ) |  |  |  |
| yaoEPF | TTTTGTGTGATGGAGATCCGTA | *yaoE* promoter | Amplification of *yoaE* promoter sequence used for construction of plasmid p19T-P*yoaE* |  |
| yoaEPR | CCCCATTCTAATTAAACGGAAG |  |  |  |

| **Primer name** | **Sequence （5'-3'）*^a^*** | **Target gene** | **Application** |  |
| --- | --- | --- | --- | --- |
| M13F (FAM) | CGCCAGGGTTTTCCCAGTCACGAC (5’labeled with FAM) | pMD-19T | Amplification of the FAM-tagged *yoaE* promoter sequence for Dnase 1 footprinting experiments using p19T-P*yoaE* as a template |  |
| M13R | AGCGGATAACAATTTCACACAGGA |  |  |  |
| cpxPF1 | CGCGATTCAACGAGAGACA | *cpxP* promoter | Amplification of FAM labeled *cpxP* promoter, used in EMSA |  |
| cpxPR1-FAM | CAGCTCTCGGTCATCATCAAC (5’labeled with FAM) |  |  |  |
| yoaEP50F | AAAATATCCCGGTTACATCTCTTTGCAAAGGAAGGTAAATCTTTGCCAAA | *yaoE* promoter | Generating unlabeled 50 bp *yoaE* promoter, used in EMSA |  |
| yoaEP50R | TTTGGCAAAGATTTACCTTCCTTTGCAAAGAGATGTAACCGGGATATTT |  |  |  |
| yoaEP50F-FAM | AAAATATCCCGGTTACATCTCTTTGCAAAGGAAGGTAAATCTTTGCCAAA (5’labeled with FAM) | *yaoE* promoter | Annealing with yoaEP50R to generate FAM labeled 50 bp *yoaE* promoter, used in EMSA |  |

*^a^*Letter with underline are homologous arm sequence immediately adjacent to the targeted region to be deleted.
